# Supplementary material for: MetaQTL: a package of new computational methods for the meta-analysis of QTL mapping experiments
Source: BMC Bioinformatics. 2007 Feb 8;8:49. doi: 10.1186/1471-2105-8-49 (PMC1808479; doi:10.1186/1471-2105-8-49)
Supplement: Additional File 7 — MetaQTL Package : jar file and tutorial. This Zip archive contains both the MetaQTL JAR file and the files of the tutorial. [file 1471-2105-8-49-S7.zip › org.inra.metaqtl/doc/org/metaqtl/algo/package-summary.html]

org.metaqtl.algo


|  |  |  |
| --- | --- | --- |
| **Overview** \| **Package** \| Class \| **Use** \| **Tree** \| **Deprecated** \| **Index** \| **Help** | |  |
| **PREV PACKAGE**   **NEXT PACKAGE** | **FRAMES**    **NO FRAMES**     **All Classes** |  |


---

## Package org.metaqtl.algo

| **Class Summary** | |
| --- | --- |
| **ClustAlgorithm** |  |
| **CMSAlgorithm** | Use this class to find common marker sequences between two chromosomes. |
| **EMAlgorithm** | This class defines methods to perform the EM-algorithm for univariate mixture of gaussians with known variances. |
| **HClustAlgorithm** | This class implements stantard agglomerative hierarchical clustering methods. |
| **MetaAlgorithm** |  |
| **MetaMapAlgorithm** | This class is dedicated to the construction of a consensus genetic map from a set of genetic maps based on a Weigthed Least Square approach. |
| **QtlClustAlgorithm** | This class defines methods to perform a QTL meta-analysis on a genetic map by applying a clustering of QTL based on a gaussian mixture model and the EM-algorihtm. |
| **QtlProjAlgorithm** | This class defines some methods to project QTL positions from a genetic map to another. |
| **QtlTreeAlgorithm** | This class defines methods to perform agglomerative hierarchical QTL clustering. |
| **SVDAlgorithm** | This class defines methods to solve usual linear algebra problems by using Singular Value Decomposition (SVD) strategy. |

---


|  |  |  |
| --- | --- | --- |
| **Overview** \| **Package** \| Class \| **Use** \| **Tree** \| **Deprecated** \| **Index** \| **Help** | |  |
| **PREV PACKAGE**   **NEXT PACKAGE** | **FRAMES**    **NO FRAMES**     **All Classes** |  |


---
